# Supplementary material for: Photodynamic Inactivation of Bacteria and Biofilms with Benzoselenadiazole-Doped Metal-Organic Frameworks
Source: Molecules. 2022 Dec 14;27(24):8908. doi: 10.3390/molecules27248908 (PMC9781904; doi:10.3390/molecules27248908)
Supplement: Supplementary file 1 [file molecules-27-08908-s001.zip › molecules-2066531-supplementary.pdf]

molecules

MDPI

Article

Photodynamic Inactivation of Bacteria and Biofilms with Benzoselenadiazole-Doped Metal-Organic Frameworks

Liang Luan [1,†], Lehan Du [2,†], Wenjun Shi [2], Yunhui Li [1,*] and Quan Zhang [2,*]

[1] Department of Laboratory Medical Center, General Hospital of Northern Theater Command, No.83, Wenhua Road, Shenhe District, Shenyang 110016, China
[2] Institute of Smart Biomedical Materials, School of Materials Science and Engineering, Zhejiang Sci-Tech University, Hangzhou 310018, China
* Correspondence: 18909882737@163.com (Y.L.); quanzhang@zstu.edu.cn (Q.Z.)
† These authors contributed equally to this work.

Citation: Luan, L.; Du, L.; Shi, W.; Li, Y.; Zhang, Q. Photodynamic Inactivation of Bacteria and Biofilms with Benzoselenadiazole-Doped Metal-Organic Frameworks. *Molecules* **2022**, *27*, x. https://doi.org/10.3390/xxxxx

Academic Editor: Peizhou Li

Received: date
Accepted: date
Published: date

Publisher's Note: MDPI stays neutral with regard to jurisdictional claims in published maps and institutional affiliations.

Copyright: © 2022 by the authors. Submitted for possible open access publication under the terms and conditions of the Creative Commons Attribution (CC BY) license (https://creativecommons.org/licenses/by/4.0/).

1. Experimental Section

Materials. Zirconium(IV) chloride ($ZrCl_4$), cesium carbonate (Cs_2CO_3), cesium fluoride (CsF), tetrakis(triphenylphosphine)palladium ($Pd(PPh_3)_4$), methyl 4-boronobenzoate, 4,7-dibromo-2,1,3-benzothiadiazole, cobalt(II) chloride hexahydrate ($CoCl_2·6H_2O$), selenium dioxide (SeO_2), [1,1′-bis(diphenylphosphino)ferrocene]dichloropalladium(II) ($Pd(dppf)Cl_2$), dichloromethane (DCM), 1,4-dioxane, ethanol (EtOH), ethyl acetate (EtOAc), methyl alcohol (MeOH), tetrahydrofuran (THF), petroleum ether (PE) and trifluoroacetic acid (TFA) were purchased from Shanghai Titan Scientific Co., Ltd. Phosphate-buffered saline was purchased from Cytiva Inc. (China). Yeast extract and tryptone were obtained from Thermo Fisher Scientific. Agar power was purchased from Shanghai EKEAR Bio&Tech Co., Ltd. Crystal violet staining solution and 4% paraformaldehyde fix solution were purchased from Beyotime Inc. (China). All other chemicals were commercially available and used without further purifications.

General Characterizations. ^{1}H NMR spectra were taken on a Bruker Advance instrument at 400 MHz. Scanning electron microscopy (SEM) images were produced using a vltra55 scanning electron microscope (CarlZeissSMTPteLtd), which operated at 3.0 KV accelerating voltage. Fourier-transform infrared spectroscopy (FT-IR) spectra were obtained as KBr-pellets on a Nicolet iS50 FT-IR spectrometer. Powder X-ray diffraction (XRD) analyses were performed and XRD patterns were recorded on a Bruker A8 Advance X-ray diffractometer using monochromatic Cu Kα radiation (λ = 1.5406 Å) at room temperature. Low-pressure gas sorption measurements were performed at 77 K using Quantachrome Instruments Autosorb-iQ (Boynton Beach, Florida USA) with the extra-high pure gases. The electron spin resonance (ESR) spectra were recorded using a Brucker A300 spectrometer at room temperature. UV-vis absorption spectra were recorded on a P7 Double Beam UV-vis spectrometer (China). Fluorescence spectra were recorded on a Hitachi F-7000 spectrophotometer. The results were presented as mean ± standard deviation (SD). Data were analyzed for statistical significant differences (*p <

0.05, **p < 0.01 and ***p < 0.001) using the SPSS software.

2. Synthesis of mTPDC-H_2 (Scheme S1)

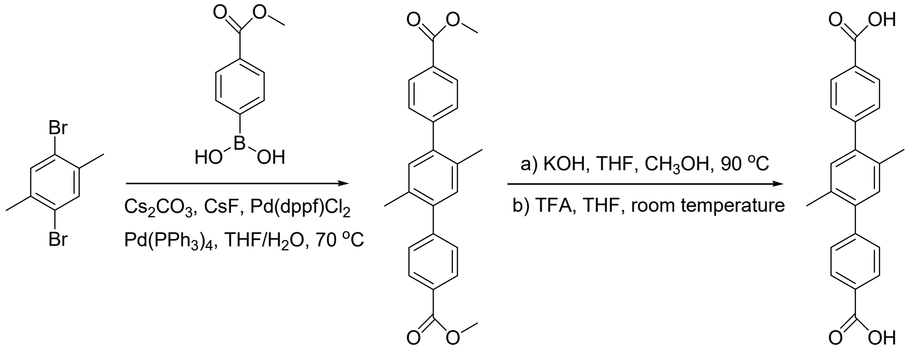

1 **2** **mTPDC-H_2**

Scheme S1. Synthetic route of mTPDC-H_2.

Cs_2CO_3 (11.1 g, 34.1 mmol) and CsF (0.86g, 5.68 mmol) were dissolved in water (2 mL). After 1,4-dioxane (100 mL) was added to the solution, the mixture was degassed by sparging with N_2 for 2 h. Then, compound ***1*** (3.0 g, 11.4 mmol), methyl 4-boronobenzoate (6.2 g, 34.1 mmol) and $Pd(PPh_3)_4$ (1.31 g, 1.14 mmol) were added into the mixture. The reaction mixture was heated to 110 °C and stirred under an argon atmosphere for 36 h. After cooling to room temperature, the reaction mixture was diluted with DCM (400 mL) and washed three times with deionized water (400 mL). The organic layer was dried over anhydrous Na_2SO_4 and the solvent was evaporated *in vacuo*. The crude product was purified by column chromatograph on silica gel (DCM/EtOAc = 1:1) to give compound **2** (3.9 g, yield: 92%). To obtain mTPDC-H2, compound **2** (3.9 g, 10.4 mmol) was dissolved in THF (100 mL) and a solution of KOH (5.8 g, 104.2 mmol) in methanol (30 mL) added. The mixture was heated to 90 °C and stirred at reflux for 12 h. After cooling to room temperature, the white solid was collected by filtration and washed three times with THF (200 mL). Then, the residue was dispersed in THF (100 mL) and TFA (10 mL) was added. After the mixture was stirred at room temperature for 10 h, the crude product was obtained by centrifugation and washed with a solution of EtOH and water (1:1) to give mTPDC-H2 (3.1 g, yield: 86%). ^1H NMR (400 MHz, DMSO-d_6, ppm) δ = 8.03 (d, J = 8.1 Hz, 4H), 7.53 (d, J = 8.2 Hz, 4H), 7.20 (s, 2H), 2.24 (s, 6H). ^{13}C NMR (101 MHz, DMSO-d_6, ppm) δ = 167.16, 145.33, 139.79, 132.31, 131.56, 129.21, 19.53.

3. Synthesis of SeTPDC-H2 (Scheme S2)

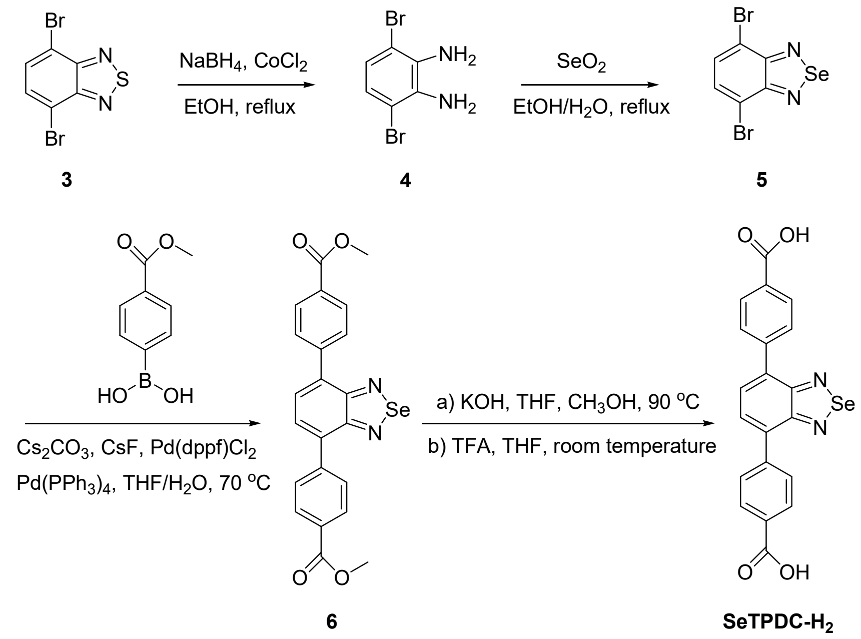

Scheme S2. Synthetic route of SeTPDC-H2.

The synthetic route of SeTPDC-H_2 is divided into four steps. First, a mixture of 4,7-dibromo-2,1,3-benzothiadiazole (5.0 g, 17.0 mmol), $NaBH_4$ (1.29 g, 34.1 mmol) and $CoCl_2{\cdot}6H_2O$ (0.41 g, 1.7 mmol) in EtOH (200 mL) was heated to 90 °C and stirred under a nitrogen atmosphere for 4 h. After cooling down to room temperature, the insoluble solid was removed by filtration. The solvent was removed under reduced pressure and the residue was extracted with DCM (400 mL). The organic layer was dried over anhydrous Na_2SO_4 and the solvent was evaporated *in vacuo*. The crude product was purified by column chromatography on silica gel (DCM/PE = 1:1) to afford compound ***4*** (4.1 g, yield: 91%).

Secondly, compound ***4***（3.0 g, 11.3 mmol）were dissolved in EtOH (90 mL) and a solution of SeO_2（1.3 g, 11.4 mmol）in hot water (50 mL) was added dropwise to the mixture. The mixture was heated to 90 °C and stirred for 12 h. After cooling to room temperature, the yellow solid was collected by filtration and washed with EtOH to give compound ***5*** (3.5 g, yield: 91%).

Thirdly, Cs_2CO_3 (10.0 g, 30.6 mmol) and CsF (0.78 g, 5.1 mmol) were dissolved in water (3 mL) and THF (100 mL) was added to the mixture. The reaction mixture was degassed by sparging with N_2 for 2 h, and then compound ***5*** (3.5 g, 10.2 mmol), methyl 4-boronobenzoate (4.2 g, 23.5 mmol), Pd(dppf)Cl_2 (0.75 g, 1.02 mmol) and Pd(PPh_3)$_4$ (0.35 g, 0.31 mmol) were added under a nitrogen atmosphere. The reaction mixture was heated to 70 °C and stirred for 48 h. After cooling down to room temperature, the mixture was

extracted two times with DCM (200 mL). The combined organic layer was washed three times with water (400 mL) and dried over anhydrous Na_2SO_4. After the solvent was removed under reduced pressure, the crude product was purified by column chromatography on silica gel (DCM/EtOAc = 50:1) to give compound ***6*** (3.7 g, yield: 80%).

Finally, compound ***6*** (0.84 g, 1.86 mmol) was dissolved in THF (100 mL) and a solution of KOH (1.10 g, 19.64 mmol) in MeOH (20 mL) was added to the mixture. The reaction mixture was heated to 90 °C and stirred at reflux for 12 h. After cooling to room temperature, the yellow solid was collected by filtration and washed three times with THF (200 mL). Then, the residue was dispersed in THF (100 mL) and TFA (10 mL) was added. After the mixture was stirred at room temperature for 10 h, the crude product was obtained by centrifugation and washed three times with a solution of EtOH and water (1:1) to give SeTPDC-H2 (0.65 g, yield: 83%). ^{1}H NMR (400 MHz, DMSO-d_6, ppm) δ = 13.04 (s, 2H), 8.08 (s, 8H), 7.85 (s, 2H). ^{13}C NMR (101 MHz, DMSO-d_6, ppm) δ = 167.13, 158.31, 141.79, 133.66, 130.16, 129.62, 129.24, 128.56.

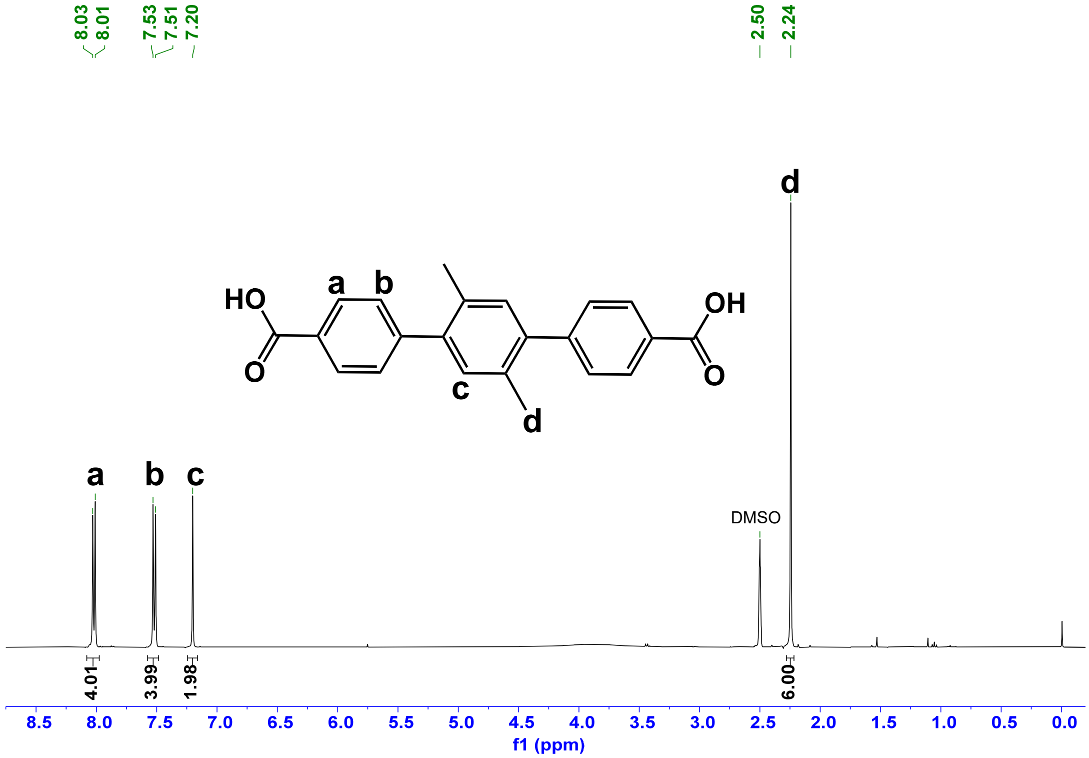

Figure S1. ^{1}H NMR spectrum of mTPDC-H_2 in DMSO-d_6.

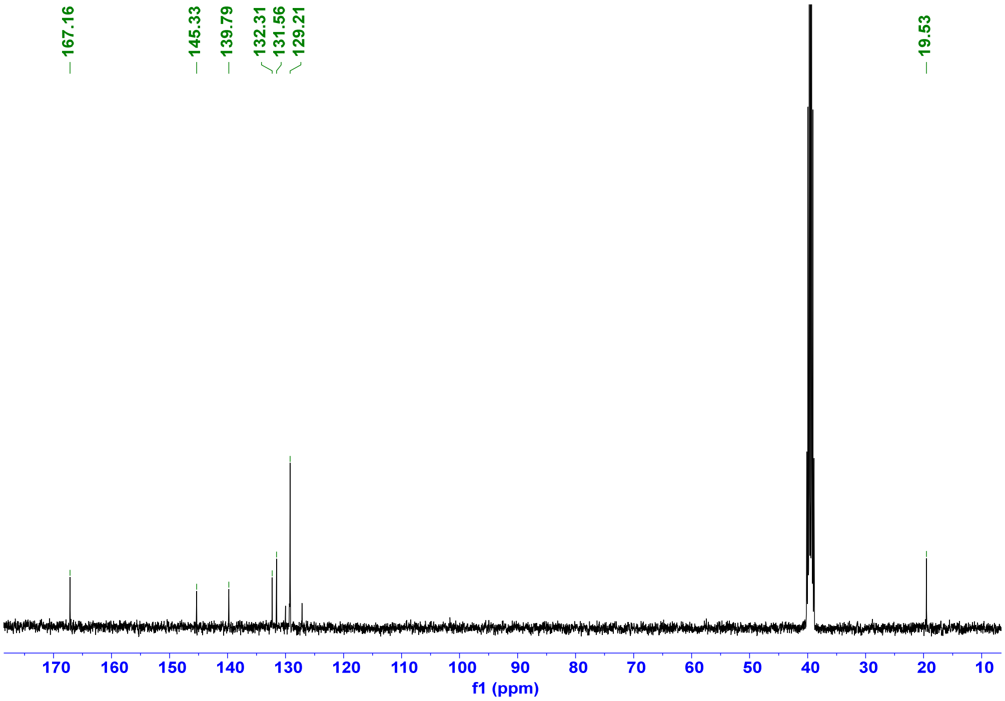

Figure S2. ^{13}C NMR spectrum of mTPDC-H_2 in DMSO-d_6.

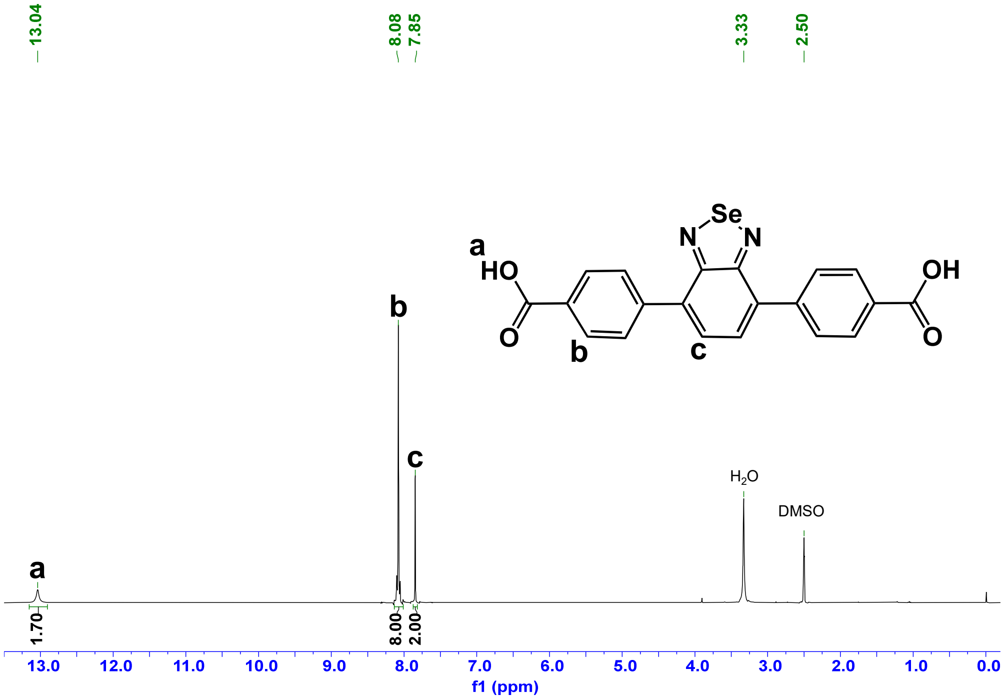

Figure S3. ^{1}H NMR spectrum of SeTPDC-H2 in DMSO-d_6.

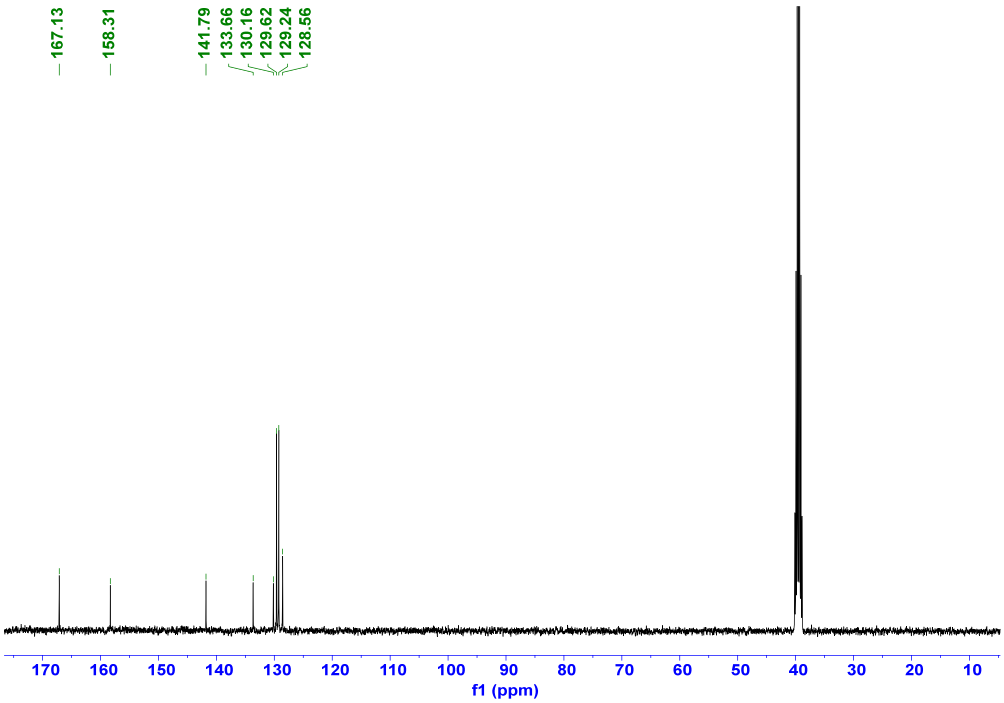

Figure S4. ^{13}C NMR spectrum of SeTPDC-H_2 in DMSO-d_6.
